# Patients with metastatic renal cell carcinoma who receive immune-targeted therapy may derive survival benefit from nephrectomy

**DOI:** 10.1186/s12885-023-11408-x

**Published:** 2023-10-06

**Authors:** Hanzhi Dong, Yuan Cao, Yan Jian, Jun Lei, Weimin Zhou, Xiaoling Yu, Xiquan Zhang, Zhiqiang Peng, Zhe Sun

**Affiliations:** 1 Department of Oncology, The First Affiliated Hospital of Nanchang University, Nanchang, 330006 China; 2 Department of Medical Oncology, Jiangxi Cancer Hospital, The Second Affiliated Hospital of Nanchang Medical College, Jiangxi Clinical Research Center for Cancer,, Nanchang, 330029 China; 3 Department of Oncology, Jiangxi Provincial People’s Hospital, The First Affiliated Hospital of Nanchang Medical College, Nanchang, 330006 China; 4 Department of Urology, Jiangxi Cancer Hospital, The Second Affiliated Hospital of Nanchang Medical College, Jiangxi Clinical Research Center for Cancer, Nanchang, 330029 China; 5 Department of Oncology, Yugan Xinjiang Hospital, Shangrao, 335100 China; 6 Department of Lymphohematology, Jiangxi Cancer Hospital, The Second Affiliated Hospital of Nanchang Medical College, Jiangxi Clinical Research Center for Cancer, Nanchang, 330029 China

**Keywords:** Nephrectomy, Immunotherapy, Targeted therapy, Renal cell carcinoma

## Abstract

**Background:**

Nephrectomy, whether in the era of cytokine therapy or targeted therapy, has an important role in the treatment of metastatic renal cell carcinoma. With the advent of immunotherapy, immunotherapy combined with targeted therapy has become the mainstream of systemic therapy, but the role of nephrectomy in metastatic renal cell carcinoma is unclear. In this study, we retrospectively analyzed the impact of nephrectomy on survival in patients with metastatic renal cell carcinoma who received immune-targeted therapy.

**Methods:**

Patients with metastatic renal cell carcinoma who received immune-targeted therapy at three centers between May 17, 2019 and August 1, 2022 were collected, who were divided into two groups based on whether nephrectomy was performed or not. Survival, response rate and adverse event were compared between the two groups. The primary end point was progression free survival, Subgroup analysis and univariate and multivariable prognostic analyses were also assessed.

**Results:**

With a median follow-up time of 29.3 months (95% CI 28.5–30.2), 165 patients were recruited and divided into two groups based on whether they underwent nephrectomy or not. There were 68 patients in the non-nephrectomy group, 97 in the nephrectomy group. Compared to patients treated with immune-targeted therapy, patients treated with immune-targeted therapy plus nephrectomy were able to achieve survival benefits, with a median PFS of 10.8 months (95% CI 8.3–13.3) and 14.4 months (95% CI 12.6–16.2), respectively, as well as an HR of 0.476 (95% CI 0.323–0.701, *p* = 0.0002). The 12-month and 18-month PFS rates were 30.9% versus 60.8% and 7.4% versus 25.8%, respectively. The objective response rate (ORR) was 52.9% and 60.8%, respectively, in the non-nephrectomy and nephrectomy groups (*p* = 0.313), and the disease control rate (DCR) was 75% and 83.5%, respectively (*p* = 0.179). The most common adverse events related to treatment were hypothyroidism, immune-related pneumonitis and rash. Multivariate analysis showed that primary tumor nephrectomy prior to immune-targeted therapy, clear cell renal carcinoma and oligo metastasis were independent prognostic factors.

**Conclusions:**

Nephrectomy may provide PFS benefit with tolerable safety for patients with metastatic renal cell carcinoma who receive immune-targeted therapy. In multivariate analysis, nephrectomy, clear cell carcinoma, and oligo-organ metastasis were found to be favorable independent prognostic factors.

**Supplementary Information:**

The online version contains supplementary material available at 10.1186/s12885-023-11408-x.

## Introduction

In recent decades, great strides have been made in the treatment of metastatic renal cell carcinoma (mRCC) through the era of cytokine therapy, targeted therapy and immunotherapy [1]. However, the morbidity and mortality of RCC continues to be high. In the United States it was estimated that there were 76,080 new cases of kidney cancer and an estimated 13,780 deaths in 2021 [2]. Diversifying treatment approaches have led to prolonged progression-free survival (PFS) and overall survival (OS). Despite this, current treatment is not sufficient to meet clinical needs. The best treatment modality has been explored, and there has been debate as to whether nephrectomy can provide survival benefit to patients.

The disappearance of lung metastases after nephrectomy has been reported as far back as 1973 [3], and similar reports have been reported since [4]. Cytoreductive nephrectomy has subsequently been more commonly attempted in advanced renal cancer. In the prospective study by Flanigan et al., the median survival was better with nephrectomy plus interferon compared with interferon alone, with a 31% lower risk of mortality [5]. However, in the era of targeted therapy, the CARMENA study found sunitinib alone to be non-inferior to nephrectomy followed by sunitinib in patients with mRCC, the media OS was 18.4 months in the sunitinib alone group compared with 13.9 months in the nephrectomy plus sunitinib group [6]. Ziad Bakouny et al. reviewed over 4000 patients with mRCC who received immunotherapy or targeted therapy and found that cytoreductive nephrectomy (CN) was only associated with significantly better OS in both the immunotherapy(HR: 0.61; 95% CI, 0.41–0.90, *p* = 0.013) and the targeted therapy (HR: 0.72; 95% CI, 0.67–0.78, *p* < 0.001) [7].

Survival benefits for primary renal cell carcinoma resection are not uniform in different systemic treatment settings. In the cytokine era, cytoreductive nephrectomy used to be the standard of care and the cornerstone for the management of mRCC. However, this trend was broken by the CARMENA study. While nephrectomy conferred no survival benefit for intermediate-and poor-risk mRCC patients, the sunitinib alone group had longer survival. Following the entry of immune checkpoint inhibitors into clinical practice, the role of nephrectomy in mRCC is more akin to that of a fog, and only retrospective clinical analysis is available [8, 9].  At present, the mainstay of treatment for mRCC is PD-1 inhibitor plus VEGFR-targeted [10], to date, no studies have evaluated the role of nephrectomy in the background of targeted therapy in combination with immunotherapy. Herein, we performed a retrospective analysis of patients with metastatic renal cell carcinoma who received combined immune and targeted therapy at three centers, grouped according to whether or not they had undergone nephrectomy, and assessed the survival, efficacy and safety.

## Methods

### Study design and patients

We conducted a multicenter, retrospective clinical study in The First Affiliated Hospital of Nanchang University, Jiangxi Cancer Hospital and Jiangxi Provincial People’s Hospital from May 17, 2019 to August 1, 2022 in Nanchang, China. This retrospective, multicenter study was approved by the Ethics Committees of above three hospitals.

**Fig. 1:**
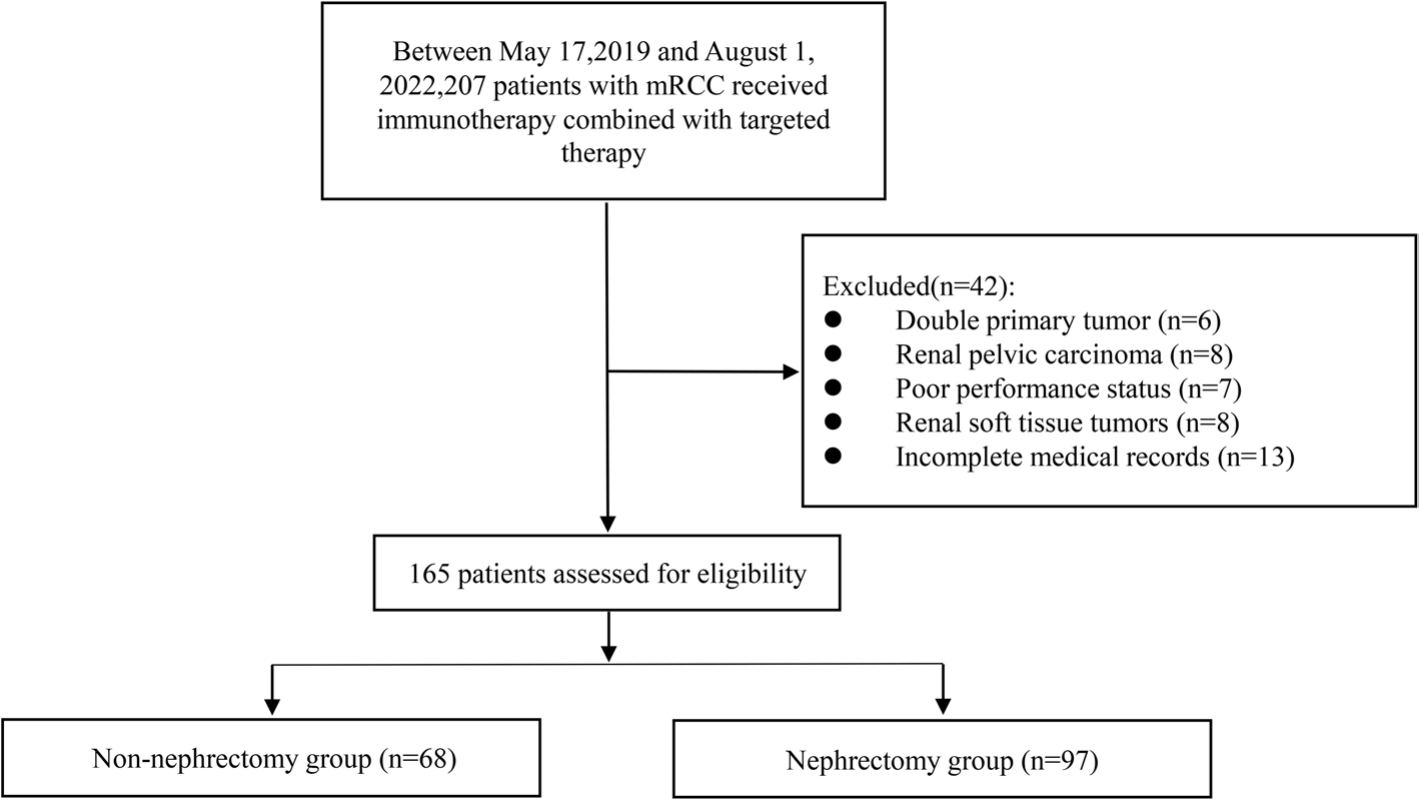
Patient flow chart

After medical record review, we screened 207 patients with metastatic renal cell carcinoma who were receiving a combination of targeted and immunotherapy at three centers.42 patients were excluded, including 6 with double primary tumors, 8 with renal pelvic carcinoma, 7 with poor performance status, 8 with renal soft tissue tumors and 13 with incomplete medical records. A total of 165 eligible patients were divided into two groups based on nephrectomy history: the non-nephrectomy group were 68 patients, 97 patients in the nephrectomy group were defined as either radical nephrectomy (58 patients) or cytoreductive nephrectomy (39 patients) prior to immune-targeted therapy (Fig. 1). The progression-free survival (PFS) was the primary study endpoint, objective response rate (ORR) and adverse event (AE) were secondary study endpoints, OS maturity was 46.7% only, further follow-up was needed, and the final follow-up was October 29, 2022.

Inclusion criteria were as follows: (1) Age > 18 years; (2) Pathologic diagnosis of renal cell carcinoma; (3) Advanced or recurrent renal cell carcinoma with measurable lesions; (4) Patients were treated with combination targeted and immunotherapy; (5) Patient’s lifetime expectancy > 1 month; (6) Eastern Cooperative Oncology Group performance status from 0 to 2. Exclusion criteria were as follows: (1) Dual primary tumors; (2) Renal uroepithelial carcinoma or renal soft tissue tumor; (3) Dual immunotherapy; (4) Incomplete medical records.

### Systematic treatment

All patients received targeted combination immunotherapy at least 2 cycles. The information of immunotherapy and targeted therapy was in the Supplementary table 1 and 2.

### Assessments and follow-up

Patient data were obtained from the electronic medical record systems at the three centers, ensuring data authenticity and traceability of the data. Monitoring strategies were consistent across the three centers. Baseline imaging was defined as less than 1 month prior to immune-targeted treatment. Contrast-enhanced computed tomography (CT) or magnetic resonance imaging (MRI) should be performed to evaluate the response. Following immune-targeted therapy, response was assessed every 2 cycles based on Response Evaluation Criteria in Solid Tumors 1.1 (RECIST 1.1). Adverse events (AE) were evaluated according to the National Cancer Institute’s Common Terminology Criteria version 5.0 (CTCAE 5.0) up to 60 days after immune-targeted therapy. Time to first treatment, disease progression, last follow-up and death were recorded.

### Statistical analysis

Categorical variables were tested by chi-square test and continuous variables were tested by one-way ANOVA. Categorical variables were described by frequencies or percentages, and continuous variables were described by medians and ranges. PFS and OS were analyzed by Kaplan‒Meier method. Cox regression was used for subgroup analysis and univariate multivariate analysis, the hazard ratio and 95% confidence interval (95% CI) were calculated. Statistical analyses were performed using SPSS version 28.0 (IBM) and GraphPad Prism version 9.4.1 (GraphPad, Inc.). A 2-tailed *P* value<0.05 was considered statistically significant for all data analyses.

## Result

### Patient characteristics

We screened 207 patients with metastatic renal cell carcinoma from 3 centers. Participants met the following inclusion and exclusion criteria. A total of 165 patients were enrolled and divided into two groups according to whether they underwent nephrectomy or not. There were 68 patients in the non-nephrectomy group, 97 in the nephrectomy group. The median age of patients in both groups was 59 years, with more than 80% of patients having PS = 1 and clear cell carcinoma. Nearly 50% of the patients were WHO/ISUP grade 2, and more than 60% of the patients were IMDC grade moderate risk. The majority of patients had normal baseline kidney function. Immune-targeted therapy was used as first-line therapy in 63.2% of the patients in the non-nephrectomy group and 55.7% of the patients in the nephrectomy group, fewer than 4% of patients in both groups were treated with more than 4-line regimens, and nearly 50% of patients selected axitinib as their target therapy. In general, Baseline demographics and Disease-Related Information for all patients were balanced across the two groups (Table 1).

**Table 1:** Baseline demographics and disease-related information for all patients

Characteristic	Non-nephrectomy (*n* = 68) (%)	Nephrectomy (*n* = 97) (%)	*P* value
Age, y	59	59	0.421
Range	19–86	26–84	
Sex			0.631
Male	46 (67.6)	69 (71.1)	
Female	22 (32.4)	28 (28.9)	
ECOG performance status			0.174
0	5 (7.4)	7 (7.2)	
1	55 (80.9)	86 (88.7)	
2	8 (11.8)	4 (4.1)	
BMI, kg/m^2^			0.098
Greater than or equal to 24	12 (17.6)	28 (28.9)	
Less than 24	56 (82.4)	69 (71.1)	
Histopathology			0.846
Clear cell carcinoma	56 (82.4)	81 (83.5)	
Non-clear cell carcinoma	12 (17.6)	16 (16.5)	
WHO/ISUP grade			0.367
1	13 (19.1)	16 (16.5)	
2	32 (47.1)	48 (49.5)	
3	21 (30.9)	24 (24.7)	
4	2 (2.9)	9 (9.3)	
IMDC grade			0.078
Low risk	3 (4.4)	15 (15.5)	
Moderate risk	47 (69.1)	61 (62.9)	
Poor risk	18 (26.5)	21 (21.6)	
Primary renal tumor size (Maximum Diameter, cm) (95%CI)	6.5 (5.9–7.1)	6.6 (6.1–7.2)	0.753
Clinical T stage			0.917
T1	31 (45.6)	45 (46.4)	
T2	16 (23.5)	23 (23.7)	
T3	19 (27.9)	20 (20.6)	
T4	2 (3.0)	9 (9.3)	
Hypertension			0.533
No	48 (70.6)	64 (66.0)	
Yes	20 (29.4)	33 (34.0)	
Smoking history			0.103
Never smoker	48 (70.6)	79 (81.4)	
Former or Current smoker	20 (29.4)	18 (18.6)	
Radiotherapy history			0.154
No	62 (91.2)	81 (83.5)	
Yes	6 (8.8)	16 (16.5)	
Baseline renal function			0.147
Normal	52 (76.5)	64 (66.0)	
Abnormal	16 (23.5)	33 (34.0)	
Immune-targeted therapy line			0.725
1	43 (63.2)	54 (55.7)	
2	15 (22.1)	29 (29.9)	
3	8 (11.8)	11 (11.3)	
More than or equal to 4	2 (2.9)	3 (3.1)	
Combination targeted agent			0.158
Axitinib	30 (44.1)	44 (45.4)	
Lenvatinib	11 (16.2)	6 (6.2)	
Anlotinib	8 (11.8)	10 (10.3)	
Other	19 (27.9)	37 (38.1)	

### Survival analysis

At the last follow-up, with a median follow-up time of 29.3 months (95% CI 28.5–30.2). 56 of 68 (82.4%) patients in the non-nephrectomy group and 80 of 97 (82.5%) patients in the nephrectomy group were found to have disease progression. A total of 77 of 165 patients died, with 34 of 68 (50%) patients in the non-nephrectomy group and 43 of 97 (44.3%) patients in the nephrectomy group, resulting in an overall survival maturity of only 46.7%, all patients died due to progressive disease.

**Fig. 2:**
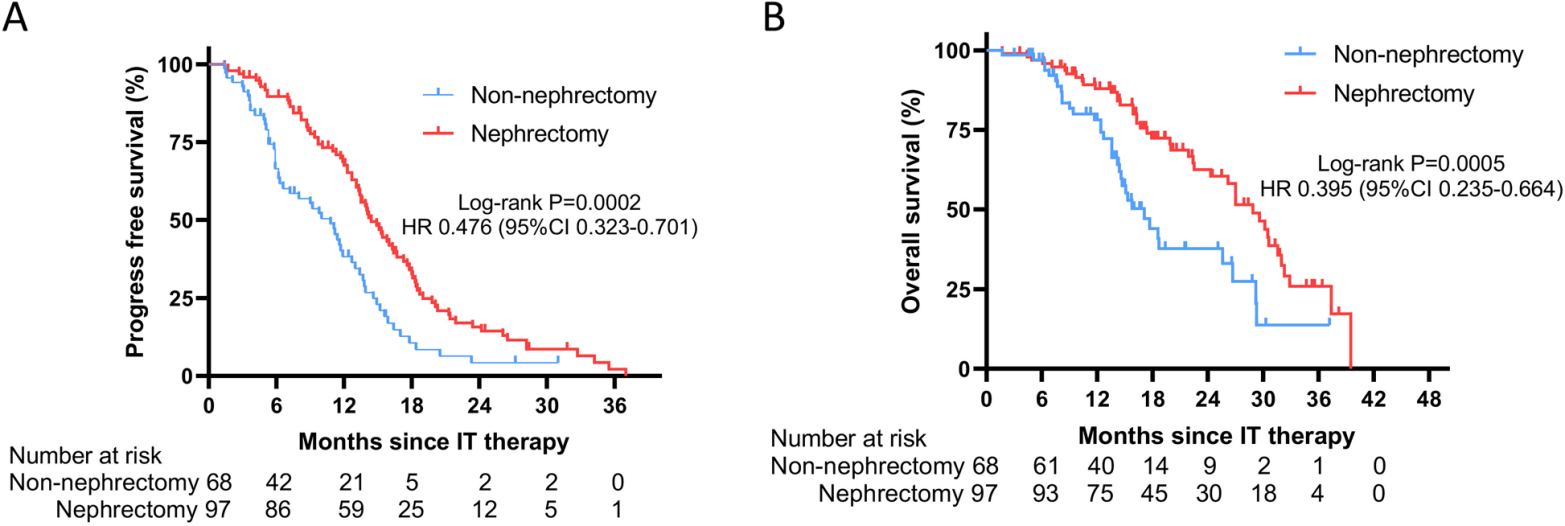
Kaplan–Meier curves of survival outcomes of patients in the two groups. **A** Comparison of progression free survival between two groups. **B** Comparison of overall survival between two groups

Compared to patients treated with immune-targeted therapy, patients treated with immune-targeted therapy plus nephrectomy were able to achieve survival benefits, with a median PFS of 10.8 months (95% CI 8.3–13.3) and 14.4 months (95% CI 12.6–16.2), respectively, as well as an HR of 0.476 (95% CI 0.323–0.701, *p* = 0.0002). The risk of disease progression was reduced by 52.4% in the nephrectomy group. The 12-month and 18-month PFS rates in the non-nephrectomy and nephrectomy groups were 30.9% versus 60.8% and 7.4% versus 25.8%, respectively (Fig. 2 A).

More than half of patients failed to reach OS. Preliminary OS data showed that patients in the nephrectomy group had a significantly better OS than those in the non-nephrectomy group, with a median overall survival of 28.9 months (95%CI 25.2–32.6) compared to 17.1 months (95%CI 13.7–20.5), respectively, and an HR of 0.395 (95%CI 0.235–0.664). The 12-month and 18-month OS rates were 58.8% versus 77.3% and 20.6% versus 46.4% in the non-nephrectomy and nephrectomy groups, respectively (Fig. 2 B).

**Fig. 3:**
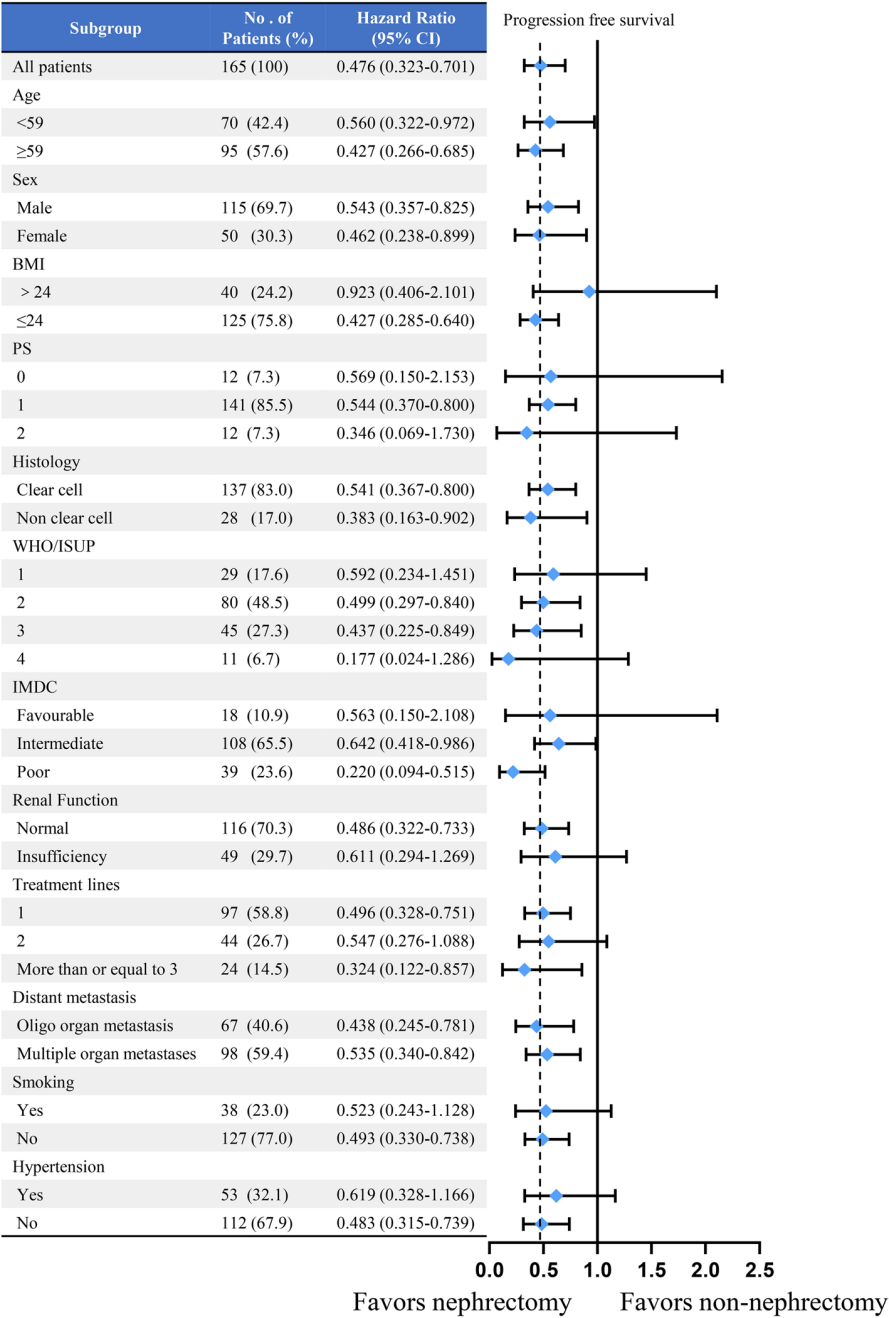
Forest plot for progression free survival of the matched cohorts of patients

Since maturity of OS was less than 50%, we performed subgroup analysis for PFS only. In all subgroups, the prognosis was better after nephrectomy prior to immune-targeted therapy, especially in patients with WHO/ISUP 4 and IMDC poor risk, with HR around 0.2 (Fig. 3).

### Efficacy

One patient achieved complete response (CR) in the nephrectomy group. The objective response rate (ORR) was 52.9% and 60.8%, respectively, in the non-nephrectomy and nephrectomy groups (*p* = 0.313), and the disease control rate (DCR) was 75% and 83.5%, respectively (*p* = 0.179). Although there was no significant difference in efficacy between the two groups, the ORR and DCR were higher in the nephrectomy group compared to the group that did not undergo nephrectomy (Table 2).

**Table 2:** Summary of best response

Variable	Non-nephrectomy (*n* = 68) (%)	Nephrectomy (*n* = 97) (%)	*P* value
Complete response	0 (0.0)	1 (1.0)	
Partial response	36 (52.9)	58 (59.8)	
Stable disease	15 (22.1)	22 (22.7)	
Progressive disease	17 (25.0)	16 (16.5)	
Objective response rate	52.9	60.8	0.313
Disease control rate	75.0	83.5	0.179

### Safety and tolerability

Although most patients tolerated immune-targeted therapy, only one patient discontinued immunotherapy due to immune myocarditis. The most common adverse events related to treatment were hypothyroidism, immune-related pneumonitis and rash. The incidence of drug-related kidney injury was higher in the nephrectomy group than in the non-nephrectomy group. In the nephrectomy group, there was a 7.4% incidence of grade 3/4 adverse events, including hypothyroidism, drug-related kidney injury, drug-related liver injury, pneumonia and myocarditis (Table 3).

**Table 3:** Treatment-related adverse events

Adverse events	Any Grade			3/4 Grade		
	**Non-nephrectomy (*n* = 68) (%)**	**Nephrectomy (*n* = 97) (%)**	***P* value**	**Non-nephrectomy (*n* = 68) (%)**	**Nephrectomy (*n* = 97) (%)**	***P* value**
**Treatment-related AEs, n (%)**						
Rash	6 (8.8)	14 (14.4)	0.28	0 (0.0)	0 (0.0)	
Hypothyroidism	15 (22.1)	18 (18.6)	0.58	1 (1.5)	2 (2.1)	0.78
Hyperthyroid	3 (4.4)	4 (4.1)	0.93	0 (0.0)	0 (0.0)	
Drug-induced kidney injury	6 (8.8)	16 (16.5)	0.15	1 (1.5)	0 (0.0)	0.23
Pneumonia	9 (13.2)	7 (7.2)	0.20	2 (2.9)	2 (2.1)	0.72
Drug-induced liver injury	2 (2.9)	10 (10.3)	0.07	1 (1.5)	0 (0.0)	0.23
Gastrointestinal reaction	2 (2.9)	4 (4.1)	0.69	0 (0.0)	0 (0.0)	
Myelosuppression	2 (2.9)	2 (2.1)	0.72	0 (0.0)	0 (0.0)	
Myocarditis	0 (0.0)	1 (1.0)	0.40	0 (0.0)	1 (1.0)	0.40
Eye Toxicity	0 (0.0)	0 (0.0)		0 (0.0)	0 (0.0)	
Skeletal muscle toxicity	0 (0.0)	0 (0.0)		0 (0.0)	0 (0.0)	
Injection reaction	0 (0.0)	0 (0.0)		0 (0.0)	0 (0.0)	
Neurotoxicity	0 (0.0)	0 (0.0)		0 (0.0)	0 (0.0)	

### Prognostic factor analysis

Univariate and multivariate prognostic factor analyses for progression-free survival are summarized in Table 4. Multivariate analysis showed that primary tumor nephrectomy prior to immune-targeted therapy, clear cell renal carcinoma and oligo organ metastasis (Supplementary table 3) were independent prognostic factors for metastatic renal cell carcinoma patients receiving immune-targeted therapy, and these patients would have better PFS.

**Table 4:** Univariate and multivariate analysis of risk factors for progression-free survival

Variables	Progression-free survival					
	**Univariate analysis**			**Multivariate analysis**		
	**HR**	**95%CI**	***P* value**	**HR**	**95%CI**	***P* value**
Nephrectomy (yes/no)	0.488	0.337–0.707	< 0.001	0.499	0.349–0.713	< 0.001
Age (y), (< / ≥ 59)	1.038	0.723–1.491	0.839			
Sex, (male/female)	0.855	0.563–1.298	0.462			
BMI (kg/m^2^), (> / ≤ 24)	1.562	1.008–2.422	0.046			
PS, (0–1/2)	1.192	0.553–2.572	0.654			
Histopathology, (clear cell/non clear cell)	0.597	0.374–0.955	0.031	0.582	0.373–0.909	0.017
WHO/ISUP grade, (1–2/3–4)	1.060	0.735–1.528	0.754			
IMDC, (favorable, intermediate/poor)	0.896	0.542–1.481	0.669			
Baseline renal function, (normal/insufficiency)	0.786	0.521–1.185	0.250			
Treatment lines, (1/ ≥ 2)	1.002	0.696–1.444	0.990			
Adverse events, (no/yes)	0.667	0.458–0.971	0.035			
Distant metastasis, (oligo/multiple)	0.608	0.415–0.889	0.010	0.666	0.468–0.948	0.024
Smoking, (no/yes)	1.334	0.812–2.190	0.255			
Hypertension, (no/yes)	0.882	0.592–1.313	0.535			

## Discussion

In a pooled analysis of the SWOG and EORTC studies, cytoreductive nephrectomy combined with cytokines was confirmed to be able to prolong the overall survival of patients, compared with cytokine therapy alone [5, 11, 12]. Cytoreductive nephrectomy was the standard of care for metastatic renal cell carcinoma before the era of targeted therapy. Several retrospective studies have suggested that in the era of targeted therapy, there may be survival benefit to patients from cytoreductive nephrectomy. In a retrospective study analysis, 314 patients were recruited, 201 patients undergoing CN and 113 patients not undergoing CN, and observed a median OS of 19.8 months versus 9.4 months [13]. Subsequently, similar conclusions have been reached in other retrospective clinical studies, in the SEER database, and in the National Cancer database with large-sample data analysis [14–16]. However, CARMENA study performed a prospective multicenter clinical study in patients with metastatic clear cell carcinoma, and concluded that monotherapy with sunitinib was non-inferior to nephrectomy plus sunitinib for overall survival, especially for patients at intermediate and poor risk for MSKCC score [6]. SURTIME study in the same year explored the systemic therapy of sunitinib and the timing of nephrectomy, and delayed cytoreductive nephrectomy was found not to improve the 28-week progression-free rate (PFR), meanwhile, patients with delayed cytoreductive nephrectomy had a longer OS [17]. Both studies were prospective, randomized controlled trials, but the subtype of patients was clear cell carcinoma only. In CARMENA study, only intermediate and poor risk patients were enrolled, and no favorable risk patients were available. Only 99 patients were enrolled in the SURTIME study, which was far below the estimated sample size. Therefore, both studies have some limitations, and their reference for clinical application is limited.

Vascular endothelial growth factor (VEGF) promotes immune suppression by enhancing the influx of suppressive cell types into the tumor microenvironment (TME), modulating the activity of myeloid-derived suppressor cells (MDSCs) and Treg cells, inhibiting the maturation of dendritic cells (DCs) [18, 19]. Small molecule TKI plays an anti-tumor role by inhibiting VEGF receptor to reduce tumor angiogenesis and immunosuppression. However, only treatment of mRCC with TKIs was prone to drug resistance, and PFS was only approximately 6 to 11 months [20, 21]. Immune checkpoint inhibitors block the binding of immunosuppressive molecules to restore anti-tumor responses. In preclinical theory, TKIs in combination with immune checkpoint inhibitors were reported to have a synergistic anti-tumor effect [22]. KEYNOTE 426 and CLEAR studies later confirmed that immunotherapy in combination with targeted therapy was significantly superior to targeted therapy alone [23, 24]. Immunotherapy combined with tyrosine kinase inhibitors (TKIs) was approved by the FDA for the treatment of mRCC in 2019 [25].  As the types of systemic therapy change, the re-evaluation of the role of nephrectomy in mRCC is important.

In this study, we retrospectively analyzed 165 patients with mRCC treated with immunotherapy in combination with targeted therapy. Patients were divided into two groups according to whether they underwent nephrectomy or non-nephrectomy prior to combined immune-targeted therapy. However, it is important to note that the median PFS in the nephrectomy group was 3.6 months longer than that in the non-nephrectomy group, and an HR of 0.476 (95% CI 0.323–0.701, *p* = 0.0002), the 12- and 18-month PFS rates in the nephrectomy group were significantly higher than those in the non-nephrectomy group. Preliminary OS analysis suggested that the overall survival of the nephrectomy group was significantly better than that of the non-nephrectomy group, although the ORR and DCR in the nephrectomy group were slightly higher than those in the non-nephrectomy group, but the difference did not reach statistically significant. The two groups were comparable in terms of safety. Multivariate analysis showed that nephrectomy, clear cell carcinoma and oligo-organ metastasis were independent favorable prognostic factors for mRCC with immune-targeted therapy.

The human immune system is also a double-edged sword for tumors, the immune system not only inhibit tumor growth but also promote tumor progression by alterations in gene mutations, tumor microenvironment, and cell signaling pathways [26]. TCGA database transcriptome analysis identified that clear cell renal cell carcinoma enriched immune infiltration and T cell infiltration [27]. Studies have confirmed that the immune components and functions of primary and metastatic renal cell carcinoma roughly overlap [28], however, the higher expression of PD-L1 and a lower CD8 to Foxp3 T cell ratio were found in metastatic lesions compared with matched primary tumor tissue [29]. Several hypotheses explained the potential mechanisms by which nephrectomy confers survival benefits. First, increased apoptosis of lymphocytes [30], impairment of T-cell receptors and signal transduction [31], and dysfunction of tumor-infiltrating lymphocytes [32] were observed in renal cell carcinoma tissues. Primary renal cell carcinoma has also been shown to produce high levels of proinflammatory cytokines and T-cell inhibitory cytokines such as interleukin 8 (IL-8), IL-6, transforming growth factors, and weaken the immune response [33]. Second, Myeloid cells lead to tumor progression and metastasis by promoting angiogenesis and vasculogenesis as well as inhibiting anti-tumor immunity. Accumulation of tolerogenic DCs and the elevated levels of circulating MDSCs in RCC are detrimental to the immune microenvironment and worsen the metastatic lesions burden [34–36]. Moreover, the expression of immune response target factors, such as CTLA-4,B7-H1,B7-H3,B7-H4 and PD-1 on the surface of tumor cells and effector T cells can be negatively regulated by these immunosuppressive factors and cells, resulting in tumor cells immune escape [37, 38]. Therefore, primary renal tumor resection reduces the burden of immunosuppressive factors and cells, enhances the immune response.

Renal cell carcinomas are heterogeneous tumors with different immunogenicity characteristics. Clear cell renal cell carcinoma (ccRCC) are characterized by rich leukocyte infiltrates, which often consist of CD8^+^T cells, CD4^+^ T cells, natural killer cells and myeloid cells [39, 40]. High proliferative activity CD8 + T cells reflect excellent antitumor immunity and are associated with longer survival [41]. Thompson et al. first reported the expression of PD-L1 in ccRCC, and 66.7% of 196 clear cell carcinoma samples had PD-L1 expression >  = 5% [38]. In another study, tumor cell PD-L1 expression was found to be 10.9% in 101 non-ccRCC patients [42]. Although PD-L1 positive tumors are associated with a worse clinicopathological classification, such advanced TNM stage and higher Fuhrman grade, they are also one of potential biomarkers for immunotherapy [43]. A phase II trial of cabozantinib plus nivolumab in patients with non-ccRCC found that the objective response rate for patients with papillary, unclassified, or translocation-associated RCC (*n* = 40) was 47.5%, with mPFS 12.5 months, in patients with chromophobe RCC (*n* = 7), no response was observed [44]. Patients enrolled in KEYNOTE 426 were clear cell renal cell carcinoma, with an ORR of 59.3% and an mPFS of 15.1 m. Analysis of existing clinical research data suggests that the efficacy and survival of patients with clear cell renal cell carcinoma who receive combination targeted immunotherapy is superior to that of non-ccRCC. The ccRCC group had better PFS than the non-ccRCC group in this study, which may be related to tumor-infiltrating T lymphocytes activity and PD-L1 expression. The factors that affect the efficacy of immune-targeted combination therapy are complex, including immune evasion, metabolic reprogramming and the immune microenvironment in addition to TILS and PD-L1 [45, 46]. Thus, the efficacy of immunotherapy in combination with targeted therapy in both clear cell carcinoma and non-clear cell carcinoma requires further investigation.

We recognize the limitations of non-randomized, retrospective, analyses. Data were obtained from three centers, but the sample size was limited and OS maturity was lacking, with over half of patients failing to progress to OS. Further, there was heterogeneity between the two groups of patients with combination immune-targeted drugs, although the characteristics of the two groups were balanced. Depth of response was not analyzed, due to limited data. Outcomes would benefit from longer follow-up.

## Conclusion

Nephrectomy may provide PFS benefit with tolerable safety for patients with advanced renal cell carcinoma who receive immune-targeted therapy. In multivariate analysis, nephrectomy, clear cell carcinoma, and oligo-organ metastasis were found to be favorable independent prognostic factors.

### Supplementary Information

**Additional file 1: Table 1.** Category and dosage of target medicine and PD-1 inhibitors. **Table 2.** Two groups of PD-1 inhibitors combined with targeted therapy. **Table 3.** Distribution of oligometastasis in the two groups.

## Data Availability

The datasets used and/or analyzed during the current study available from the corresponding author on reasonable request.
