# Supplementary material for: Patients with metastatic renal cell carcinoma who receive immune-targeted therapy may derive survival benefit from nephrectomy
Source: BMC Cancer. 2023 Oct 6;23:943. doi: 10.1186/s12885-023-11408-x (PMC10557339; doi:10.1186/s12885-023-11408-x)
Supplement: Supplementary file 1 — Additional file 1: Table 1. Category and dosage of target medicine and PD-1 inhibitors. Table 2. Two groups of PD-1 inhibitors combined with targeted therapy. Table 3. Distribution of oligometastasis in the two groups. [file 12885_2023_11408_MOESM1_ESM.docx]

Patients with advanced renal cell carcinoma who receive immune-targeted therapy may derive survival benefit from nephrectomy

Hanzhi Dong^a,1^, Yuan Cao^b,1^, Yan Jian ^a,1^, Jun Lei^c^, Weimin Zhou^d^, Xiaoling Yu^e^, Xiquan Zhang^c,🖂^, Zhiqiang Peng^f,🖂^ ,Zhe Sun^b,🖂^

^a^ Department of Medical Oncology, Jiangxi Cancer Hospital, The Second Affiliated Hospital of Nanchang Medical College, Jiangxi Clinical Research Center for cancer, Nanchang 330029, China.

^b^ Department of Oncology, The First Affiliated Hospital of Nanchang University, Nanchang, 330006, China

^c^ Department of Oncology, Jiangxi provincial People's Hospital, The First Affiliated Hospital of Nanchang Medical College, Nanchang,330006, China

^d^ Department of Urology, Jiangxi Cancer Hospital, The Second Affiliated Hospital of Nanchang Medical College, Jiangxi Clinical Research Center for cancer, Nanchang 330029, China.

^e^ Department of Oncology, Yugan Xinjiang Hospital, Shangrao, 335100, China

^f^ Department of Lymphohematology, Jiangxi Cancer Hospital, The Second Affiliated Hospital of Nanchang Medical College, Jiangxi Clinical Research Center for cancer, Nanchang 330029, China

^1^These authors contributed equally to this work

^🖂^**Corresponding Author:**

Zhe Sun: Department of Oncology, The First Affiliated Hospital of Nanchang University, Nanchang, 330006, China, Email: acpzq@163.com

Zhiqiang Peng: Department of Lymphohematology, Jiangxi Cancer Hospital, The Second Affiliated Hospital of Nanchang Medical College, Jiangxi Clinical Research Center for cancer, Nanchang 330029, China, Email: [ndzhlyy1277@ncu.edu.cn](mailto:ndzhlyy1277@ncu.edu.cn).

Xiquan Zhang: Department of Oncology, Jiangxi provincial People's Hospital, The First Affiliated Hospital of Nanchang Medical College, Nanchang,330006, China, Email: Zhangxiquan1243@126.com

Supplement Table

Table 1. Category and dosage of target medicine and PD-1 inhibitors

| **Category** | **Dose (mg)** | **Category** | **Dose (mg)** |
| --- | --- | --- | --- |
| Axitinib | 5 bid | Pembrolizumab | 200 |
| (Pfizer) |  | (Carlow, Merck Sharp & Dohme Corp) |  |
| Lenvatinib | 8-12 | Nivolumab | 240 |
| (Eisai China Inc) |  | (Squibb Pharma) |  |
| Anlotinib | 8-12 | Toripalimab | 240 |
| (Tian qing Inc) |  | (Suzhou, Hezhong pharmaceutical Co.Ltd) |  |
| Bevacizumab | 7.5mg/kg | Sintilimab | 200 |
| (Roche Pharma Co.Ltd) |  | (Suzhou, Xinda pharmaceutical Co.Ltd)) |  |
| Sunitinib | 50 | Camrelizumab | 200 |
| (Pfizer) |  | (Jiangsu Hengrui Medicine Co.Ltd) |  |
| Sorafenib | 400 bid |  |  |
| （Bayer AG） |  |  |  |

**Table 2. Two groups of PD-1 inhibitors combined with targeted therapy**

| **Combined regimen** | **Non-nephrectomy Arm（n=68）** | **Nephrectomy Arm (n=97)** |
| --- | --- | --- |
| Axitinib+Pembrolizumab | 6 | 8 |
| Axitinib+Toripalimab | 15 | 19 |
| Axitinib+Sintilimab | 5 | 10 |
| Axitinib+Camrelizumab | 4 | 7 |
| Lenvatinib+Pembrolizumab | 2 | 2 |
| Lenvatinib+Toripalimab | 6 | 4 |
| Lenvatinib+Sintilimab | 3 | 0 |
| Anlotinib+Nivolumab | 1 | 3 |
| Anlotinib+Toripalimab | 7 | 7 |
| Bevacizumab+Toripalimab | 1 | 4 |
| Sunitinib+Sintilimab | 9 | 17 |
| Sorafenib+Toripalimab | 9 | 16 |

**Table 3. Distribution of oligometastasis in the two groups**

| **Oligometastatic organ** | **Non-nephrectomy Arm（n=26）** | **Nephrectomy Arm (n=41)** |
| --- | --- | --- |
| lung | 11 | 22 |
| bone | 7 | 5 |
| liver | 2 | 2 |
| Lymph nodes | 3 | 4 |
| others | 3 | 8 |
